# Generalized Trust and Intelligence in the United States

**DOI:** 10.1371/journal.pone.0091786

**Published:** 2014-03-11

**Authors:** Noah Carl, Francesco C. Billari

**Affiliations:** Department of Sociology and Nuffield College, University of Oxford, Oxford, United Kingdom; Hunter College, City University of New York (CUNY), CUNY School of Public Health, United States of America

## Abstract

Generalized trust refers to trust in other members of society; it may be distinguished from particularized trust, which corresponds to trust in the family and close friends. An extensive empirical literature has established that generalized trust is an important aspect of civic culture. It has been linked to a variety of positive outcomes at the individual level, such as entrepreneurship, volunteering, self-rated health, and happiness. However, two recent studies have found that it is highly correlated with intelligence, which raises the possibility that the other relationships in which it has been implicated may be spurious. Here we replicate the association between intelligence and generalized trust in a large, nationally representative sample of U.S. adults. We also show that, after adjusting for intelligence, generalized trust continues to be strongly associated with both self-rated health and happiness. In the context of substantial variation across countries, these results bolster the view that generalized trust is a valuable social resource, not only for the individual but for the wider society as well.

## Introduction

The idea that trust in other members of society facilitates commerce and exchange goes back at least to Adam Smith [1], who noted that a merchant often prefers to trade within his own country because there “he can know better the character and situation of the persons whom he trusts.” Consistent with Smith’s intuition, an extensive empirical literature has established that generalized trust is an important aspect of civic culture–or social capital as it is also known [2]–[5]. Research has found, for example, that countries whose citizens place greater trust in one another have more efficient public institutions [2], [3] and experience higher rates of economic growth [4], [5]. In addition, research has found that individuals who place greater trust in their fellow citizens are more likely to start a business [6], perform voluntary work more often [7], report better physical health [8], and claim to be happier with their lives overall [9]. The study of generalized trust therefore has profound implications for public policy, as well as being of inherent scientific interest [2].

In social surveys, generalized trust is typically assessed with the question, “Generally speaking would you say that most people can be trusted or that you can’t be too careful in dealing with people?” The number of response categories and the precise wording of the question vary from one survey to another. Two of the most well-studied correlates of generalized trust are self-rated health and happiness. Like generalized trust, they are measured using simple survey items. Self-rated health and happiness have been shown to constitute valid proxies for physical health and evaluated well-being, respectively [10], [11]. Furthermore, the finding that people who trust others are happier and enjoy better self-rated health has been made in a diverse array of societies, using a range of statistical techniques [8], [9], [12]–[15].

Two recent studies have documented a strong correlation between generalized trust and intelligence [16], [17]. Sturgis et al. analyse data from the U.K., and show that intelligence at age 10–11 predicts generalized trust at age 34, even after conditioning on a large number of socio-economic variables, including self-rated health and happiness. Similarly, Hooghe et al. examine Dutch data, and find that a large part of the association between generalized trust and education is accounted for by cognitive ability. These results raise the possibility that previous studies have overestimated the effect of generalized trust on outcomes such as self-rated health and happiness, due to omitted variable bias. Indeed, the latest evidence from the U.K. suggests not just that more intelligent people are happier, but that the relationship between IQ and happiness is partly mediated by self-rated health [18].

## Methods

### Data

The data we analyse are from the General Social Survey (GSS), a public opinion survey that has been administered to a nationally-representative sample of U.S. adults every 1–2 years since 1972. The GSS contains questions on respondents’ socio-economic characteristics, behaviours, and social attitudes. It has been used in the past to analyse both generalized trust [4], [19]–[20] and intelligence [21]–[23]. However, the present study is the first to have used it to analyse the relationship between generalized trust and intelligence. Extensive documentation about the GSS, including the entire GSS codebook [24], can be downloaded for free at the National Opinion Research Centre’s website.

### Measures

Our first measure of intelligence is a 10-word vocabulary test in which the respondent is asked to identify which of five phrases supplies the correct definition of a given word [24]. Despite its brevity, the test has a correlation of 0.71 with the Army General Classification Test, an IQ exam developed by the U.S. Military [25]. In addition, there is abundant psychometric evidence that individuals with higher IQs have larger vocabularies [26], [27]. Prior to taking the vocabulary test, the respondent is told the following by the interviewer [24]: “We would like to know something about how people go about guessing words they do not know. On this card are listed some words–you may know some of them, and you may not know quite a few of them. On each line the first word is in capital letters–like BEAST. Then there are five other words. Tell me the number of the word that comes closest to the meaning of the word in capital letters. For example, if the word in capital letters is BEAST, you would say “4” since “animal” come closer to BEAST than any of the other words. If you wish, I will read the words to you. These words are difficult for almost everyone–just give me your best guess if you are not sure of the answer.” The respondent is assigned a score between 0 and 10, corresponding to the number of words she defined correctly.

Our second measure of intelligence is an assessment by the interviewer of how well the respondent understood the survey questions. The interviewer notes down whether the respondent’s understanding of the survey questions was “good”, “fair” or “poor” [24]. We refer to our first measure as ‘verbal ability’ and our second measure as ‘question comprehension’. The Pearson correlation between these two variables is 0.37 (*p*<0.001), meaning that they are moderately positively correlated. The strength of their correlation is depicted in Table 1, a simple cross-tabulation. It indicates that 98% of those scoring 10 out of 10 in the vocabulary test have a good understanding of the survey questions, yet only 36% of those scoring 0 out of 10 have a good understanding of them.

**Table 1:** Cross-tabulation of verbal ability by question comprehension.

	Question comprehension		
Verbal ability	Good	Fair	Poor
0	35.8	46.4	17.9
1	34.4	44.0	21.6
2	45.5	41.7	12.9
3	59.1	33.7	7.2
4	71.2	25.0	3.8
5	81.0	17.7	1.4
6	87.7	11.5	0.8
7	91.9	7.7	0.4
8	94.5	5.4	0.2
9	95.2	4.0	0.8
10	97.8	2.2	0.0

*Notes:* Values are row percentages. All respondents for whom data were available were included. *n* = 26,649.

Generalized trust is assessed with the question [24]: “Generally speaking, would you say that most people can be trusted or that you can’t be too careful in dealing with people?” The respondent may answer “can trust”, “cannot trust” or “it depends”. Consistent with previous studies, we convert generalized trust into a binary variable by assigning the value ‘1’ to all respondents who answered “can trust”, and assigning the value ‘0’ to all those who answered “cannot trust” or “it depends”. Self-rated health is assessed with the question [24]: “Would you say your own health, in general, is excellent, good, fair, or poor?” The respondent may answer “excellent”, “good”, “fair” or “poor”. We convert self-rated health into a binary variable by assigning the value ‘1’ to all respondents who answered “excellent” or “good”, and assigning the value ‘0’ to all those who answered “fair” or “poor”. Happiness is assessed with question [24]: “Taken all together, how would you say things are these days–would you say that you are very happy, pretty happy, or not too happy?” The respondent may answer “very happy”, “pretty happy” or “not too happy”. Once again, we convert happiness into a binary variable by assigning the value ‘1’ to all respondents who answered “very happy”, and assigning the value ‘0’ to all those who answered “pretty happy” or “not too happy”. (Our main results are not sensitive to whether we treat self-rated health and happiness as binary variables or quasi-continuous variables.).

Our control variables comprise: gender, age, age squared, race, language, highest level of educational attainment, marital status and log of household income. We also control for region fixed-effects and wave fixed-effects. The GSS distinguishes between three racial categories: “white”, “black” and “other”. It distinguishes between five levels of educational attainment: “less than high school”, “high school”, “junior college”, “bachelor” and “graduate”. It distinguishes between five marital statuses: “married”, “widowed”, “divorced”, “separated” and “never married”. Log of family income is the natural log of a respondent’s family income, given in constant 1986 dollars [28]. Beginning in 2006, the GSS began to sample Spanish speakers (*n* = 513), alongside English speakers. Language is a dummy variable equal to 1 if the interview was conducted in Spanish. The GSS recognises nine U.S. regions: “New England”, “Middle Atlantic”, “East North Central”, “West North Central”, “South Atlantic”, “East South Central”, “West South Central”, “Mountain” and “Pacific”. It comprises 29 waves in total. However, the number of wave dummies included in our models varies depending on the number of waves for which data on our variables were available.

### Weights

The GSS’s sampling scheme is designed to give each household an equal probability of selection, meaning that the GSS sample is self-weighting for household-level variables [24]. However, because only one interview is conducted within each household, individuals living in larger households have a lower probability of selection. This bias can be compensated for by weighting each interview in proportion to the number of adults in the household [29]. Specifically, one can utilise the variable ADULTS, which is included in the GSS data file, when estimating statistical models. Furthermore, the 2004 and 2006 waves of the GSS employed non-respondent sub-sampling [24]. There are several alternative variables one can utilise in order to compensate for this bias. We make use of the variable WTSALL, which takes into account not only the sub-sampling of non-respondents in the 2004 and 2006 waves, but also the number of adults per household in all waves of the survey. In particular, all of our models are weighted by WTSALL. (Our main results are not sensitive to whether we weight our models.).

We intentionally exclude a relatively small number of respondents from our analyses. In 1982 and 1987, black respondents were oversampled as part of a National Science Foundation research project [24]. Consequently, the 1982 and 1987 samples are not nationally representative of the U.S. population. We therefore exclude all the oversampled respondents (*n* = 707) from our analyses. (Our main results are not sensitive to whether these respondents are excluded.).

## Results

**Figure 1:**
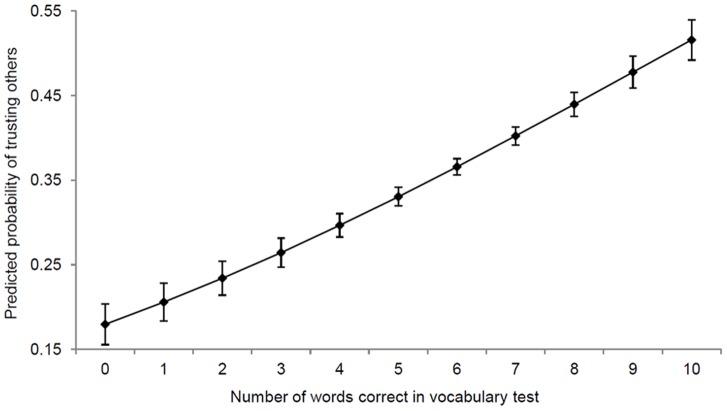
Predicted probability of trusting others by verbal ability. Predicted probabilities were estimated using a weighted probit model of generalized trust, with all other covariates held at their means. Other covariates comprise: gender, age, age squared, race, language, education, marital status, log of real household income, region fixed-effects and wave fixed-effects. Bars denote 95% confidence intervals, which were calculated using robust standard-errors. Generalized trust (*y*-axis) is a binary variable equal to 1 if the respondent “can trust others” and equal to 0 if she “cannot trust others” or if “it depends”. Verbal ability (*x*-axis) is entered as a continuous variable. Black respondents oversampled in 1982 and 1987 (*n* = 707) were excluded from the analysis. After exclusions, *n* = 13,568.

**Figure 2:**
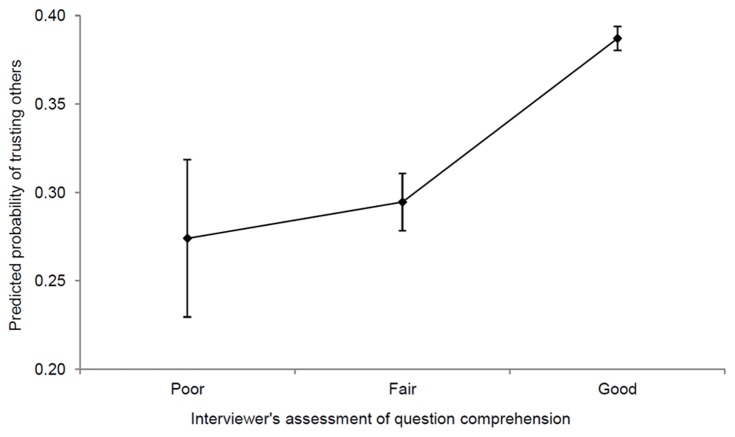
Predicted probability of trusting others by question comprehension. Predicted probabilities were estimated using a weighted probit model of generalized trust, with all other covariates held at their means. Other covariates comprise: gender, age, age squared, race, language, education, marital status, log of real household income, region fixed-effects and wave fixed-effects. Bars denote 95% confidence intervals, which were calculated using robust standard-errors. Generalized trust (*y*-axis) is a binary variable equal to 1 if the respondent “can trust others” and equal to 0 if she “cannot trust others” or if “it depends”. Question comprehension (*x*-axis) is entered as a set of binary variables. Black respondents oversampled in 1982 and 1987 were excluded from the analysis. After exclusions, *n* = 32,982.

Fig. 1 plots the relationship between generalized trust and verbal ability, conditional on socio-economic characteristics. The association between the two variables is strong and positive. An individual with the highest verbal ability is 34 percentage-points more likely to trust others than an individual with the lowest verbal ability. Fig. 2 plots the conditional relationship between generalized trust and question comprehension. Once again, the association is positive. An individual with a good understanding of the survey questions is 11 percentage-points more likely to trust others than an individual with a poor understanding of them. Both associations are robust to controlling for parents’ education, spouse’s education, and several indicators of socio-economic position at age 16 (Tables 2–3). Furthermore, the relationship holds among both men and women, among both blacks and whites, among the young, the middle-aged and the old, and in all five decades since the GSS began (Tables 4–11).

**Table 2:** Marginal effects of verbal ability from weighted probit models of generalized trust.

	Dependent variable: Generalized trust indicator		
	(1)	(2)	(3)
Verbal ability	0.061***	0.036***	0.035***
	(0.002)	(0.003)	(0.005)
Parents’ educations			yes
Spouse’s education			yes
Socio-economic resources at age 16			yes
Individual controls		yes	yes
Region and wave fixed-effects		yes	yes
Observations	14,999	13,568	4,435

*Notes*: Marginal effects were estimated with all other covariates held at their means. Robust standard-errors are reported in parentheses. Significance levels: ^†^10%, *5%, **1%, ***0.1%. Individual controls: gender, age, age squared, race dummies, a language dummy, education dummies, marital status dummies and log of real household income. Black respondents oversampled in 1982 and 1987 were excluded.

**Table 3:** Marginal effects of question comprehension from weighted probit models of generalized trust.

	Dependent variable: Generalized trust indicator		
	(1)	(2)	(3)
Fair understanding of questions	0.067***	0.020	–0.005
	(0.015)	(0.024)	(0.048)
Good understanding of questions	0.236***	0.113***	0.105*
	(0.014)	(0.023)	(0.047)
Parents’ educations			yes
Spouse’s education			yes
Socio-economic resources at age 16			yes
Individual controls		yes	yes
Region and wave fixed-effects		yes	yes
Observations	36,759	32,982	11,163

*Notes*: Marginal effects were estimated with all other covariates held at their means. Robust standard-errors are reported in parentheses. Significance levels: ^†^10%, *5%, **1%, ***0.1%. Individual controls: gender, age, age squared, race dummies, a language dummy, education dummies, marital status dummies and log of real household income. Black respondents oversampled in 1982 and 1987 were excluded.

**Table 4:** Marginal effects of verbal ability from weighted probit models of generalized trust for men and women.

	Dependent variable: Generalized trust indicator	
	Men	Women
Verbal ability	0.025***	0.046***
	(0.004)	(0.004)
Individual controls	yes	yes
Region and wave fixed-effects	yes	yes
Observations	5,973	7,595

*Notes*: Marginal effects were estimated with all other covariates held at their means. Robust standard-errors are reported in parentheses. Significance levels: ^†^10%, *5%, **1%, ***0.1%. Individual controls: age, age-squared, race dummies, a language dummy, education dummies, marital status dummies and log of real household income. Black respondents oversampled in 1982 and 1987 were excluded.

**Table 5:** Marginal effects of question comprehension from weighted probit models of generalized trust for men and women.

	Dependent variable: Generalized trust indicator	
	Men	Women
Fair understanding of questions	0.028	0.016
	(0.038)	(0.029)
Good understanding of questions	0.118**	0.110***
	(0.037)	(0.028)
Individual controls	yes	yes
Region and wave fixed-effects	yes	yes
Observations	15,008	17,974

*Notes*: Marginal effects were estimated with all other covariates held at their means. Robust standard-errors are reported in parentheses. Significance levels: ^†^10%, *5%, **1%, ***0.1%. Individual controls: age, age squared, race dummies, a language dummy, education dummies, marital status dummies and log of real household income. Black respondents oversampled in 1982 and 1987 were excluded.

**Table 6:** Marginal effects of verbal ability from weighted probit models of generalized trust for blacks and whites.

	Dependent variable: Generalized trust indicator	
	Blacks	Whites
Verbal ability	0.012*	0.041***
	(0.006)	(0.003)
Individual controls	yes	yes
Region and wave fixed-effects	yes	yes
Observations	1,602	11,316

*Notes*: Marginal effects were estimated with all other covariates held at their means. Robust standard-errors are reported in parentheses. Significance levels: ^†^10%, *5%, **1%, ***0.1%. Individual controls: gender, age, age-squared, a language dummy, education dummies, marital status dummies and log of real household income. Black respondents oversampled in 1982 and 1987 were excluded.

**Table 7:** Marginal effects of question comprehension from weighted probit models of generalized trust for blacks and whites.

	Dependent variable: Generalized trust indicator	
	Blacks	Whites
Fair understanding of questions	–0.056	0.046^†^
	(0.039)	(0.027)
Good understanding of questions	–0.030	0.157***
	(0.039)	(0.026)
Individual controls	yes	yes
Region and wave fixed-effects	yes	yes
Observations	4,073	27,297

*Notes*: Marginal effects were estimated with all other covariates held at their means. Robust standard-errors are reported in parentheses. Significance levels: ^†^10%, *5%, **1%, ***0.1%. Individual controls: gender, age, age-squared, a language dummy, education dummies, marital status dummies and log of real household income. Black respondents oversampled in 1982 and 1987 were excluded.

**Table 8:** Marginal effects of verbal ability from weighted probit models of generalized trust for different age-groups.

	Dependent variable: Generalized trust indicator		
	18–35	36–50	51–89
Verbal ability	0.036***	0.035***	0.036***
	(0.005)	(0.005)	(0.004)
Individual controls	yes	yes	yes
Region and wave fixed-effects	yes	yes	yes
Observations	4,961	3,923	4,684

*Notes*: Marginal effects were estimated with all other covariates held at their means. Robust standard-errors are reported in parentheses. Significance levels: ^†^10%, *5%, **1%, ***0.1%. Individual controls: gender, race dummies, a language dummy, education dummies, marital status dummies and log of real household income. Black respondents oversampled in 1982 and 1987 were excluded.

**Table 9:** Marginal effects of question comprehension from weighted probit models of generalized trust for different age-groups.

	Dependent variable: Generalized trust indicator		
	18–35	36–50	51–89
Fair understanding of questions	–0.042	–0.008	0.068*
	(0.050)	(0.049)	(0.031)
Good understanding of questions	0.041	0.094*	0.153***
	(0.048)	(0.047)	(0.030)
Individual controls	yes	yes	yes
Region and wave fixed-effects	yes	yes	yes
Observations	11,619	9,608	11,755

*Notes*: Marginal effects were estimated with all other covariates held at their means. Robust standard-errors are reported in parentheses. Significance levels: ^†^10%, *5%, **1%, ***0.1%. Individual controls: gender, race dummies, a language dummy, education dummies, marital status dummies and log of real household income. Black respondents oversampled in 1982 and 1987 were excluded.

**Table 10:** Marginal effects of verbal ability from weighted probit models of generalized trust for different decades.

	Dependent variable: Generalized trust indicator				
	1970s	1980s	1990s	2000s	2010s
Verbal ability	0.049***	0.045***	0.030***	0.027***	0.030**
	(0.006)	(0.005)	(0.005)	(0.007)	(0.009)
Individual controls	yes	yes	yes	yes	yes
Region fixed-effects	yes	yes	yes	yes	yes
Observations	2,723	3,460	4,081	2,065	1,239

*Notes*: Marginal effects were estimated with all other covariates held at their means. Robust standard-errors are reported in parentheses. Significance levels: ^†^10%, *5%, **1%, ***0.1%. Individual controls: gender, age, age squared, race dummies, a language dummy, education dummies, marital status dummies and log of real household income. Black respondents oversampled in 1982 and 1987 were excluded. Survey waves in 1970s: 1976, 1978. Survey waves in 1980s: 1984, 1987, 1988, 1989. Survey waves in 1990s: 1990, 1991, 1993, 1994, 1996, 1998. Survey waves in 2000s: 2000, 2006, 2008. Survey waves in 2010s: 2010, 2012.

**Table 11:** Marginal effects of question comprehension from weighted probit models of generalized trust for different decades.

	Dependent variable: Generalized trust indicator				
	1970s	1980s	1990s	2000s	2010s
Fair understanding	0.084^†^	–0.028	–0.017	0.095*	–0.083
	(0.047)	(0.045)	(0.048)	(0.047)	(0.107)
Good understanding	0.190***	0.091*	0.056	0.169***	–0.039
	(0.046)	(0.044)	(0.046)	(0.045)	(0.103)
Individual controls	yes	yes	yes	yes	yes
Region fixed-effects	yes	yes	yes	yes	yes
Observations	6,957	7,768	8,108	7,764	2,385

*Notes*: Marginal effects were estimated with all other covariates held at their means. Robust standard-errors are reported in parentheses. Significance levels: ^†^10%, *5%, **1%, ***0.1%. Individual controls: gender, age, age squared, race dummies, a language dummy, education dummies, marital status dummies and log of real household income. Black respondents oversampled in 1982 and 1987 were excluded. Survey waves in 1970s: 1972, 1973, 1975, 1976, 1978. Survey waves in 1980s: 1980, 1983, 1984, 1986, 1987, 1988, 1989. Survey waves in 1990s: 1990, 1991, 1993, 1994, 1996, 1998. Survey waves in 2000s: 2000, 2002, 2004, 2006, 2008. Survey waves in 2010s: 2010, 2012.

Why is there such a strong correlation between generalized trust and intelligence? One explanation is that intelligent individuals are better at evaluating others’ trustworthiness, meaning that they tend to select into relationships with people who are unlikely to betray their trust [30], [31]. Another possible explanation is that intelligent individuals are less likely to trust people to do things that someone being trusted might have a strong incentive not to do (e.g., repay a large sum of money). In other words, they may be better at identifying when any particular person would be likely to act untrustworthily, based on the characteristics of the prospective interaction (e.g., material payoffs, discount rates). Alternatively, it may simply be that intelligent individuals have a greater chance of interacting with people who are materially better-off, and who therefore have less to gain from acting untrustworthily. However, this seems quite implausible given that the relationship is robust to controlling for a great many different indicators of socio-economic position.

**Figure 3:**
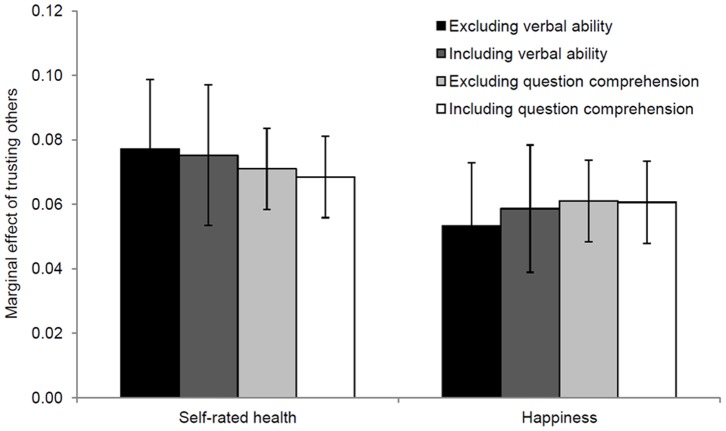
Marginal effects of trusting others on self-rated health and happiness. Marginal effects of generalized trust were estimated using weighted probit models, with all other covariates held at their means. Other covariates comprise: gender, age, age squared, race, language, education, marital status, log of real household income, region fixed-effects and wave fixed-effects. Bars denote 95% confidence intervals, which were calculated using robust standard-errors. Self-rated health is a binary variable equal to 1 if the respondent reports “good” or “excellent” health and equal to 0 if she reports “fair” or “poor” health. Happiness is a binary variable equal to 1 if the respondent is “very happy” and equal to 0 if she is “pretty happy” or “not too happy”. Generalized trust is a binary variable equal to 1 if the respondent “can trust others” and equal to 0 if she “cannot trust others” or if “it depends”. Verbal ability is entered as a continuous variable. Question comprehension is entered as a set of binary variables. Black respondents oversampled in 1982 and 1987 were excluded from the analyses. Models of self-rated health with and without verbal ability: *n* = 6,648 after exclusions. Models of self-rated health with and without question comprehension: *n* = 22,187 after exclusions. Models of happiness with and without verbal ability: *n* = 13,463 after exclusions. Models of happiness with and without question comprehension: *n* = 31,562 after exclusions.

Fig. 3 displays the conditional effects of generalized trust on self-rated health and happiness, respectively, with and without controls for intelligence. In every model, the effect of generalized trust is positive and highly significant, which is in keeping with the prior literature [8], [9], [12]–[15]. Individuals who trust others are approximately 7 percentage-points more likely to report good or excellent health, as opposed to fair or poor health. And they are approximately 6 percentage-points more likely to be very happy, rather than pretty happy or not too happy. Furthermore, controlling for intelligence does not change the strength of generalized trust’s effect on either self-rated health or happiness. The point-estimate of generalized trust’s effect on self-rated health is 3% lower when controlling for verbal ability, and is 4% lower when controlling for question comprehension. The point-estimate of generalized trust’s effect on happiness is 11% higher when controlling for verbal ability, and is <1% lower when controlling for question comprehension. In all four comparisons, the confidence intervals overlap substantially. These results provide compelling evidence that the effects of generalized trust on self-rated health and happiness are not due to confounding by intelligence.

## Robustness Checks

### Models of Generalized Trust

Table 2 displays marginal effects of verbal ability from three weighted probit models of generalized trust. In every model, the marginal effect of verbal ability is positive and highly significant (*p*<0.001). Model 1 does not include any controls. The estimate from this model implies that the probability of trusting others increases by 6 percentage-points for each correctly-defined word in the vocabulary test, holding other covariates at their means. Model 2, which includes socio-economic characteristics, region fixed-effects and wave fixed-effects, yields our preferred estimates. These are presented in Fig. 1. The estimate from model 2 implies that the probability of trusting others increases by 3.6 percentage points for each correctly-defined word in the vocabulary test, holding all other covariates at their means.

The estimate from model 3, which is approximately identical to the one from model 2, confirms that that our preferred estimates are robust to the inclusion of additional socio-economic controls, namely parents’ educations, spouse’s education, and three indicators of socio-economic resources at age 16. Parent’s educations and spouse’s education are measured the same way as the respondent’s education. Our three measures of socio-economic resources at age 16 are: type of residence at age 16, family income at age 16, and a dummy for whether the respondent was living with both of her parents at age 16. The GSS distinguishes between six different types of residence at age 16: “country non-farm”, “farm”, “town with less than 50,000 people”, “town with 50,000 to 250,000 people”, “big city suburb” and “city with more than 250,000 people”. And it distinguishes between five categories of family income at age 16, ranging from “far below average” to “far above average”. We take our preferred estimates from model 2 since these are very similar to those from model 3, yet are estimated more precisely, and are based on a substantially larger number of respondents.

Table 3 displays marginal effects of question comprehension from weighted probit models of generalized trust. The marginal effect of good question comprehension is positive and significant in all three models (*p*<0.001 in models 1 and 2, while *p*<0.05 in model 3). The estimates from model 1 imply that the probability of trusting others is 7 percentage-points higher among those with a fair understanding of the questions than among those with a poor understanding of them, and is 24 percentage-points higher among those with a good understanding, holding all other covariates at their means. Once again, model 2 yields our preferred estimates, which are presented in Fig. 2. These imply that the probability of trusting others is 2 percentage-points higher among those with a fair understanding of the questions, and is 11 percentage-points higher among those with a good understanding, holding all other covariates at their means. (The marginal effect of fair question comprehension is not significantly different from zero.) Once again, the estimates from model 3 confirm that our preferred estimates are robust to the inclusion of the additional socio-economic variables.

### Models of Generalized Trust in Subsamples

Table 4 displays marginal effects of verbal ability from weighted probit models of generalized trust for men and women. In both models, the marginal effect of verbal ability is positive and highly significant (*p*<0.001). The estimate for women is nearly twice as large as the estimate for men. Table 5 displays marginal effects of question comprehension from weighted probit models of generalized trust for men and women. In both models, the marginal effect of good question comprehension is positive and significant (*p*<0.01). The estimate for women is approximately the same size as the estimate for men.

Table 6 displays marginal effects of verbal ability from weighted probit models of generalized trust for blacks and whites. In both models, the marginal effect of verbal ability is positive and significant (*p*<0.001 in the model for whites, while *p*<0.05 in the model for blacks). The estimate for whites is substantially larger than the estimate for blacks. Table 7 displays marginal effects of question comprehension from weighted probit models of generalized trust for men and women. In the model for whites, the marginal effect of good question comprehension is positive and highly significant (*p*<0.001), yet in the model for blacks it is negative and not statistically different from zero. This is probably attributable to the small number of black respondents and the noisiness of the intelligence measure.

Table 8 displays marginal effects of verbal ability from weighted probit models of generalized trust for the young, the middle-aged and the old. In all three models, the marginal effect of verbal ability is positive and highly significant (*p*<0.001). The three estimates are approximately equal in magnitude. Table 9 displays marginal effects of question comprehension from weighted probit models of generalized trust for the young, the middle-aged and the old. In all three models, the marginal effect of good question comprehension is positive. However, while it is significant in the models for the middle-aged (*p*<0.05) and the old (*p*<0.001), it is not statistically different from zero in the model for the young. This is probably attributable to the relatively small number of young respondents and the noisiness of the intelligence measure.

Table 10 displays marginal effects of verbal ability from weighted probit models of generalized trust for each decade since the GSS began. In all five models, the marginal effect of verbal ability is positive and significant (*p*<0.01). The estimates for the three later periods are slightly lower than those for the two earlier periods. Table 11 displays marginal effects of question comprehension from weighted probit models of generalized trust for each decade since the GSS began. The marginal effect of good question comprehension is positive and significant in the models for the 1970s (*p*<0.001), the 1980s (*p*<0.05) and the 2000s (*p*<0.001). However, it is not statistically different from zero in the models for the 1990s and the 2010s. Once again, this is probably attributable to the small sample sizes and the noisy intelligence measure.

### Models of Self-rated Health and Happiness

Table 12 displays marginal effects of generalized trust from four weighted probit models of self-rated health. In every model, the marginal effect of generalized trust is positive and highly significant (*p*<0.001). Models 1 and 2 exclude and include verbal ability, respectively. The estimate from model 2 is only 3% lower than the one from model 1. Models 3 and 4 exclude and include question comprehension, respectively. The estimate from model 4 is only 4% lower than the one from model 1. Table 13 displays marginal effects of generalized trust from four weighted probit models of happiness. Once again, the marginal effect of generalized trust is positive and highly significant (*p*<0.001) in every model. The estimate from model 2, which excludes verbal ability, is 11% higher than the one from model 1, which includes verbal ability. Similarly, the estimate from model 4, which excludes question comprehension, is approximately identical to the one from model 1, which includes question comprehension. In all four comparisons the confidence intervals around the two estimates overlap considerably (Fig. 3). These results confirm that the effects of generalized trust on self-rated health and happiness are not confounded by intelligence in the GSS. They therefore strongly suggest that previous studies have not over-estimated the impact of generalized trust on health and well-being.

**Table 12:** Marginal effects of generalized trust from weighted probit models of self-rated health.

	Dependent variable: Self-rated health indicator			
	(1)	(2)	(3)	(4)
Generalized trust	0.077***	0.075***	0.071***	0.068***
	(0.011)	(0.011)	(0.006)	(0.006)
Verbal ability		yes		
Question comprehension				yes
Individual controls	yes	yes	yes	yes
Region and wave fixed-effects	yes	yes	yes	yes
Observations	6,648	6,648	22,187	22,187

*Notes*: Marginal effects were estimated with all other covariates held at their means. Robust standard-errors are reported in parentheses. Significance levels: ^†^10%, *5%, **1%, ***0.1%. Individual controls: gender, age, age squared, race dummies, a language dummy, education dummies, marital status dummies and log of real household income. Black respondents oversampled in 1982 and 1987 were excluded.

**Table 13:** Marginal effects of generalized trust from weighted probit models of happiness.

	Dependent variable: Happiness indicator			
	(1)	(2)	(3)	(4)
Generalized trust	0.053***	0.059***	0.061***	0.061***
	(0.010)	(0.010)	(0.006)	(0.006)
Verbal ability		yes		
Question comprehension				yes
Individual controls	yes	yes	yes	yes
Region and wave fixed-effects	yes	yes	yes	yes
Observations	13,463	13,463	31,562	31,562

*Notes*: Marginal effects were estimated with all other covariates held at their means. Robust standard-errors are reported in parentheses. Significance levels: ^†^10%, *5%, **1%, ***0.1%. Individual controls: gender, age, age squared, race dummies, a language dummy, education dummies, marital status dummies and log of real household income. Black respondents oversampled in 1982 and 1987 were excluded.

## Conclusion

The finding that generalized trust is highly correlated with intelligence, even after conditioning on socio-economic characteristics such as marital status, education and income, supports the hypothesis that being able to evaluate someone’s quality as a trading partner is a distinct component of human intelligence, which evolved through natural selection [30], [31]. However, there are other possible explanations for the correlation, and further research is needed to gauge the relative importance of each one. The finding that generalized trust continues to be associated with self-rated health and happiness after adjusting for intelligence reinforces the view that generalized trust is a valuable social resource–one which governments, religious groups and civic organisations should strive to cultivate [2]. Future research should focus on delineating the precise mechanisms by which generalised trust enhances people’s health and well-being.
